# Serum Procalcitonin Levels in Children with Clinical Syndromes for Targeting Antibiotic Use at an Emergency Department of a Kenyan Hospital

**DOI:** 10.1093/tropej/fmz027

**Published:** 2020-02-01

**Authors:** Samuel O. Akech, Doris W. Kinuthia, Macharia William

**Affiliations:** Department of Paediatrics and Child Health, Aga Khan University of Nairobi, P.O. Box 30270-00100, Nairobi, Kenya; Health Services Unit, Kenya Medical Research Institute/Wellcome Trust Research Programme, P.O. Box 43640-00100, Nairobi, Kenya

**Keywords:** pneumonia, sepsis, procalcitonin, children, WHO, Africa

## Abstract

Serum procalcitonin (PCT) was measured in 228 children aged 1 month to 15 years at an emergency department of a hospital located in an area without local malaria transmission in children with suspected infections; 21% (49) children had a clinical syndrome for suspected bacterial infections (Syndrome ^+ve^). In children with Syndrome^+ve^ criteria, 27/49 (55.1%) had PCT ≥ 0.5 μg/l but only 59/179 (32.9%) of those Syndrome ^+ve^ had abnormal PCT, *χ* ^2^ = 8.0, *p* = 0.005; positive likelihood ratio = 2.0 [95% confidence interval (CI) 1.2–3.3]; negative likelihood ratio = 0.8 (95% CI 0.7–1.0). In patients with pneumonia, 9/15 (60%) with severe pneumonia had PCT ≥ 0.5 μg/l compared to 11/21 (52.4%) with non-severe pneumonia, *χ* ^2^ = 0.2, *p* = 0.65. Children with clinical signs of pneumonia or clinical signs suggestive of bacterial infections fulfilling clinical syndromic definitions for suspected bacterial infections commonly have elevated PCT level. PCT levels are associated with disease severity and antibiotic trials guided by PCT levels may be needed where cultures are not available.

## Introduction

Pneumonia and other infections still cause many deaths in children aged <5 years especially in sub-Saharan Africa [1]. Clinical criteria consisting of five clinical syndromes derived from the World Health Organization (WHO) guidelines have demonstrated up to 80% sensitivity for identification of children with invasive bacterial disease in a study in Kilifi, Kenya, but had low specificity [2–5]. These syndromes include pneumonia, sick young infants (non-elective admissions aged <60 days old), meningitis/ encephalopathy, severe malnutrition and skin or soft tissue infection. The study also found the syndromic criteria mostly missed children with subtle signs of meningitis, who would be identified from results of lumbar puncture, but concluded that the criteria is valuable for identifying children with invasive bacterial disease or at risk of death. The WHO guidelines recommend antibiotic cover for all cases of childhood pneumonia diagnosed using clinical signs, both severe and non-severe [6], although a recent study from Pakistan suggests withholding antibiotics in non-severe pneumonia cases may be safe [7]. Therefore, questions are emerging whether children with pneumonia, especially non-severe cases, should all be treated with antibiotics but findings from Asia may not be generalizable to Africa because of lower overall pneumonia case fatality in Asia compared to Africa [8]. Studies have also shown viruses as important causes of pneumonia clinically indistinguishable from that caused by bacteria [9]. A recent study done in Tanzania also using a criteria derived from WHO guidance [3] showed the syndromic criteria above, with addition of shock in malaria and clinically defined sepsis, had a sensitivity of 60–70% for identification of invasive bacterial disease [4]. These criteria were developed before introduction of routine infant immunizations for *Streptoccous pneumoniae* and *Haemophilus influenzae*, which have resulted in a decline in incidence of infections from these pathogens [10], and may no longer be appropriate after the decline. Therefore, addition of pathogen diagnostics to the clinical criteria is important for targeted use of antibiotics to meet global desire to reduce antimicrobial resistance. Definitive diagnosis using culture of appropriate specimens, however, remains a problem and therefore molecular techniques and various biomarkers may be used [11] and procalcitonin (PCT) has recently emerged as a biomarker with good sensitivity, 77%, and specificity, 79% (area under the receiver operating characteristic curve 0.85) [12], for early diagnosis of sepsis [12–14] outside settings where malaria is endemic [15]. This study was done in a hospital in Nairobi, Kenya, an area without local transmission of malaria, and aimed to investigate whether children with clinically defined pneumonia or clinical syndromic criteria derived from WHO guidance, that are used to target antibiotics prescription in resource poor settings, have elevated serum PCT, a proxy marker for bacterial infection. First, changing aetiology of common disease may indicate increasing non-bacterial causes in children with clinical signs previously suggestive of bacterial infection and this study investigates whether children with syndromic criteria above have possible bacterial infection, as defined by elevated PCT. Second, we highlight the validity of PCT test in this setting and the potential need for specific microbiological diagnosis in children with suspected infections presenting to emergency settings [16].

## Methods

### Study site

The study was conducted at the outpatient department (OPD)of the Aga Khan University Hospital, a tertiary care hospital in Nairobi, Kenya. Nairobi is located at an altitude of 1795m above sea level and has no local transmission of malaria [17]. Children arriving at the OPD are first triaged by a nurse, who also measures axillary temperature. Children are then seen by a clinician on duty who takes a history, counts respiratory and heart rates, conducts a clinical assessment, and orders any laboratory or radiological investigations that are deemed necessary for making diagnosis or patient management. The clinician also decides whether to admit the child or whether care can be given as outpatient. PCT assay is available routinely at the hospital and is ordered by clinicians when they think it is clinically indicated. The OPD is run by university trained doctors (medical officers), residents specializing in paediatrics and newly qualified paediatricians. The study was conducted following scientific and ethical approval from the Aga Khan University’s Ethics and Research Committee.

### Objectives

We primarily aimed to investigate whether children with clinical diagnosis of pneumonia or with certain clinical syndromes derived from WHO guidance, which are used to target antibiotics prescriptions (sick young infants aged < 60 days, meningitis/encephalopathy, severe malnutrition and skin or soft tissue infection), have elevated PCT (≥0.5 μg/l). Children who had pneumonia or any of these signs were referred to as Syndrome^+ve^ and those without Syndrome^−ve^, and are summarized in Table 1 [4, 5]. Meningitis was clinically diagnosed and was not based on cerebrospinal fluid culture findings.

### Study participants

Clinicians at the OPD identified children aged 1 month to 15 years who had clinical signs suggestive of an infection, as defined by International Pediatric Sepsis Consensus Conference (IPSCC) [18], present for 12 or more hours. Illness had to be present for 12 or more hours because PCT level may not rise in infections with a duration <12 h According to IPSCC criteria, infections are suspected in children with systemic inflammatory response syndrome (SIRS), defined as abnormal temperature, axillary temperature >38.5°C or <36°C, and/or abnormal white blood cell count, >11 000cells/μl or <4000 cell/μl, plus either abnormal heart rate (tachycardia-heart rate > 140 bpm if aged > 12 months and > 160 bpm if aged ≤ 12 months or bradycardia-heart rate <90 if aged ≤ 12 months and < 60 bpm if aged > 12 months) or tachypnoea—> 60 bpm if ≤ 12 months and > 50 bpm if aged > 12 months. Because of delays in getting laboratory results, children were enrolled based on clinical SIRS criteria. We also modified the fever definition to include children with history of fever in current illness or those with abnormal temperature as indicated above. Children with non-infectious conditions which may present like SIRS such as trauma, burns or cancer were excluded. A study nurse stationed at the OPD was then informed by the clinician about an eligible child. The study nurse then obtained written informed consent from parent/care-giver before enrolment into the study. Participants were consecutively enrolled on weekdays during working hours.

### Sample size

This was a descriptive study conducted over a 4-month period in 2014 and sample size was not calculated.

### Study procedures

A standard medical form was used and clinical information was collected prospectively by the attending clinician at enrolment to enable classification of the participants into various clinical syndromes used to target antibiotic prescription as defined in Table 1. Assignment to various clinical syndromes was done retrospectively from clinical data entered at assessment. Pneumonia was previously classified into mild, severe and very severe pneumonia [3, 5] but we have updated the classification into only two categories, non-severe and severe pneumonia, in line with updated WHO guidance [2]. A sample for PCT (0.5 ml) was collected during routine sampling or was separately taken if there were no other tests. The study paid for costs of PCT assay. Samples were tested using BRAHMS kit (BRAHMS-Diagnostica GmbH, Hennigsdorf, Germany) following standard operating procedures using the Cobas-e^®^ immunoassay analyser. In this study, we also used other laboratory tests that were done as part of routine patient care including white cell count, platelet count, neutrophil count and any culture results.

### Data analysis

We calculated median PCT level and compared the levels in in various groups using the Kruskal-Wallis test. Geometric mean PCT levels were also calculated and compared for various groups. PCT level ≥0.5 (J.g/l is regarded as abnormal [19] and proportions with abnormal PCT were calculated and compared using the *χ* ^2^ test.

## Results

### Participant characteristics

A total of 228 out of 231 participants enrolled between July and October 2014 had PCT results therefore results described are based on the 228 participants with PCT results. Fortynine children (21.5%) fulfilled criteria for a clinical syndrome requiring antibiotic treatment (referred to as Syndrome^+ve^ criteria). This included 36 with pneumonia, 7 with clinically diagnosed meningitis/encephalopathy, 3 sick young infants, 3 with skin or soft tissue infection, and there was no case of severe malnutrition. The rest of remaining 179 participants were Syndrome^−ve^, 146 of whom had presented with history of cough or difficulty in breathing but had neither tachypnoea nor chest indrawing (no pneumonia and Syndrome^−ve^) and 33 had none of the syndromes and had no history of cough or difficulty in breathing. Eight-six children (37.7%) had PCT level ≥0.5 μg/l. Participants with Syndrome^+ve^ criteria and those Syndrome^−ve^ were similar in sex, weight and height—characteristics of participants are summarized in Table 2. Sixty-two participants had blood cultures performed and 57 of these were negative and the 5 positive cultures were reported as contaminants. Similarly, 54 participants had urine cultures done with 47 negative cultures and the 9 positive urine cultures were reported as contaminants. There were no cases of thrombocytopenia. Cerebrospinal fluid cultures were all negative in five participants where they were done.

In those with pneumonia, 21 (58.3%) were non-severe and 15 (41.7%) had severe pneumonia. A total of 135 children (59.2%) had antibiotics prescribed and 99 (43.2%) participants were admitted. Thirty-three children with history of travel to the Coastal or Western Kenya, areas of stable malaria transmission, who were tested for malaria all had negative malaria blood smears.

### PCT levels in pneumonia and WHO clinical sepsis syndromes

Overall median (interquartile range) for PCT was 0.27 (0.13–1.38) μg/l and mean ± SD was 3.87 ± 12.6 μg/l showing non-normal distribution therefore data are described using median values and geometric means. Median PCT in those with Syndrome^+ve^ criteria was 0.71 (0.18–3.99) μg/l and 0.24 (0.12–0.93) μg/l in those with Syndrome^−ve^ criteria, Kruskal–Wals, *χ* ^2^ = 7.5, *p* = 0.006. Median PCT in children with pneumonia was 0.77 (0.20–5.51) μg/l and in those without pneumonia (and Syndrome^−ve^ criteria) was 0.24 (0.12–0.78) μg/l, Kruskal-Wallis, *χ* ^2^ = 10.0, *p* = 0.002. Median value in the 13 children with other Syndrome^+ve^ criteria other than pneumonia was 0.61 (0.12–1.61) μg/l.

**Fig. 1:**
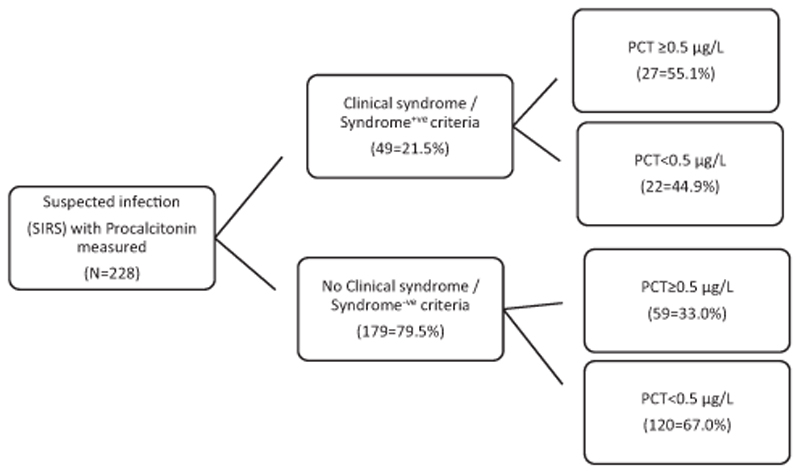
Distribution of abnormal procalcitonin (≥0.5 μg/l) by sepsis syndromes.

In children with Syndrome^+ve^ criteria, 27/49 (55.1%) had PCT ≥0.5 μg/l but only 59/179 (32.9%) of those Syndrome^−ve^ had abnormal PCT, *χ* ^2^ = 8.0, *p* = 0.005 (Fig. 1). Although presence of Syndrome^+ve^ criteria increased the likelihood of having PCT ≥0.5 μg/l, positive likelihood ratio = 2.0 [95% confidence interval (CI) 1.2–3.3], its absence Syndrome^−ve^ criteria) did not rule out abnormal PCT, negative likelihood ratio = 0.8 (95% CI 0.7–1.0). In patients with pneumonia, 9/15 (60%) with severe pneumonia had PCT ≥0.5 μg/l compared to 11/21 (52.4%) with non-severe pneumonia, *χ* ^2^ =0.2, *p* = 0.65. However, PCT ≥0.5 μg/l was present in 47/146 (32.2%) participants presenting with cough or difficulty in breathing but with no pneumonia (and Syndrome^−ve^ criteria), 7/13 (53.9%) of non-pneumonia cases who were Syndrome^+ve^, and in 12/ 33 (36.4%) of participants who were Syndrome^−ve^ without cough or difficulty in breathing.

### Procalcitonin levels vs. severity of disease

**Fig. 2:**
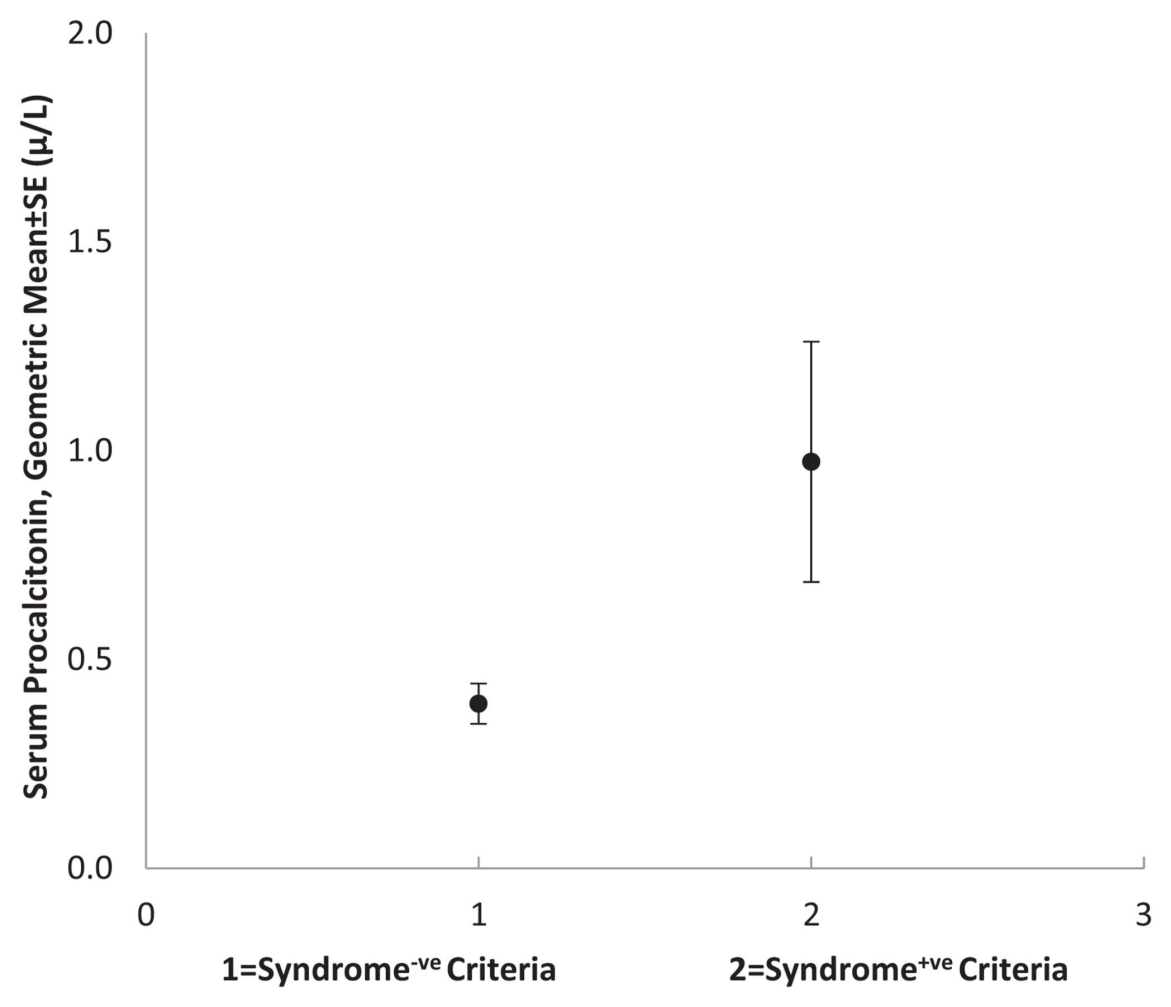
Geometric mean serum procalcitonin levels according to sepsis criteria.

**Fig. 3:**
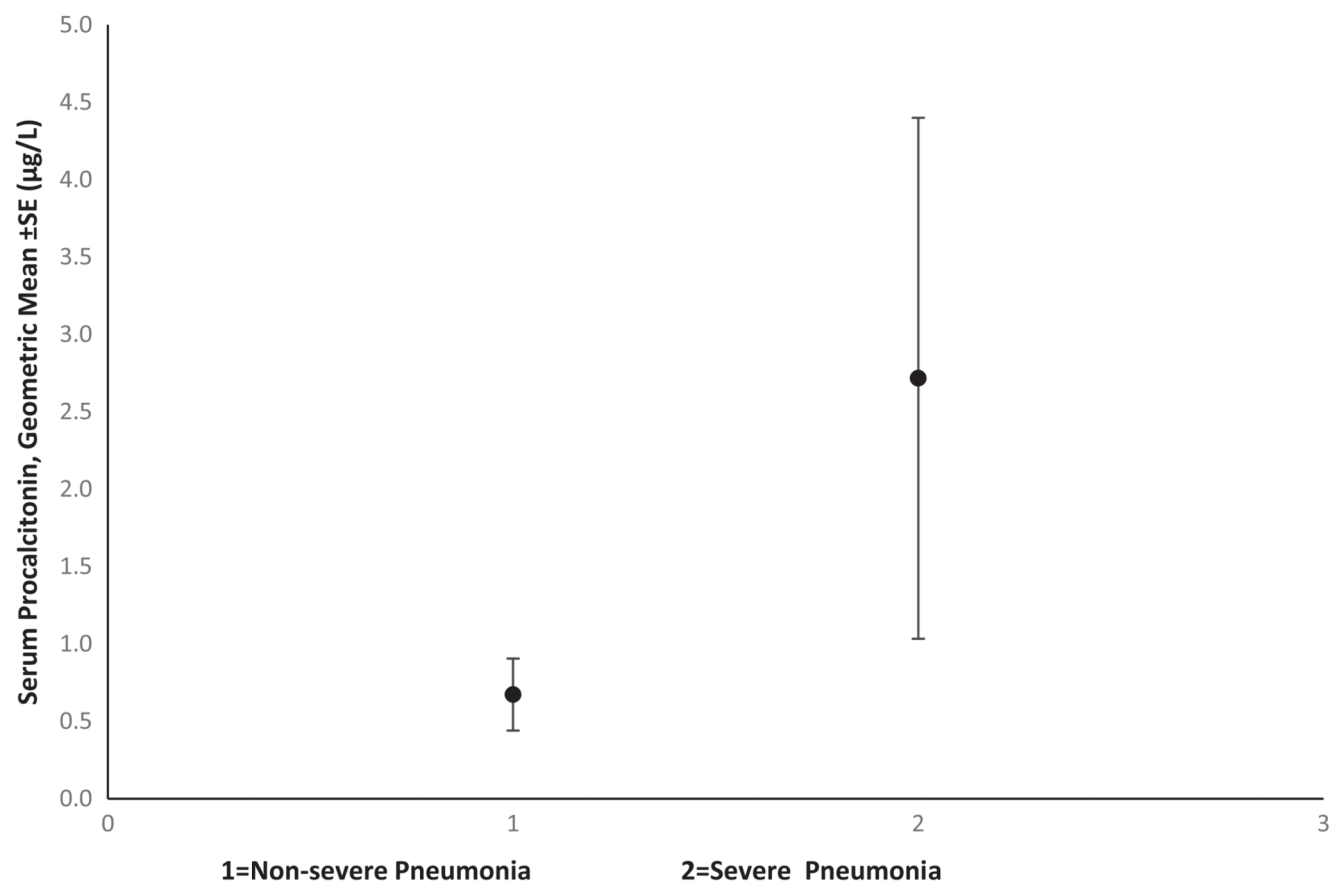
Geometric mean serum procalcitonin in severe and non-severe pneumonia. *Note*: 1 = mild pneumonia; 2 = severe pneumonia; significant difference, p = 0.007.

**Fig. 4:**
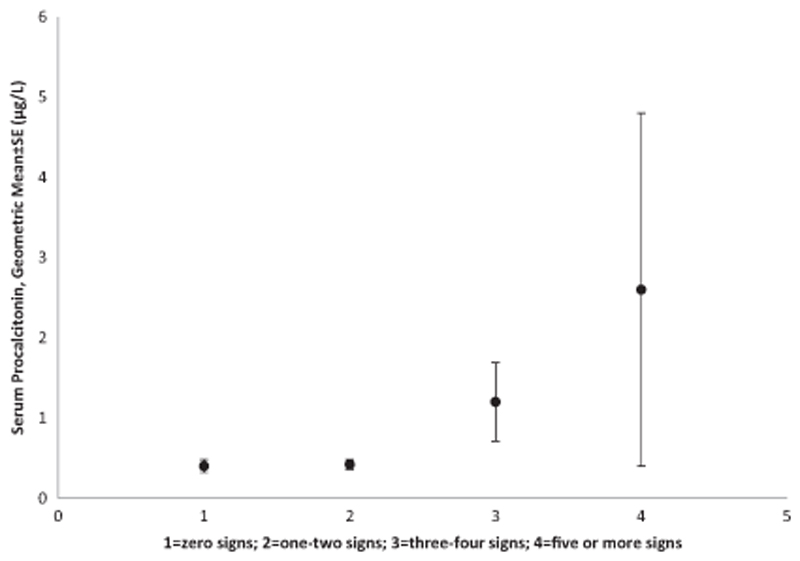
Geometric mean serum procalcitonin levels in relationship to number of clinical signs.

Median PCT in children with severe pneumonia was 1.95 (0.25–45.7) μg/l, geometric mean ± SE 2.72 ± 1.68μg/l, compared to 0.61 (0.18–3.47) μg/l, geometric mean ± SE 0.70 ± 0.23 μg/l, in non-severe pneumonia (Kruskal-Wallis, *χ* ^2^ = 2.5, *p* = 0.11)—Figs 2 and 3. When we further examined PCT levels based on the presence of any number of the following clinical signs (difficulty in breathing, convulsions, hypoxia, tachycardia, age < 60 days, central cyanosis, chest indrawing, grunting, acidotic breathing, crackles, weak pulse volume, capillary refill time >2 s, sunken eyes, delayed skin pinch, consciousness level is not alert, inability to drink or breastfeed, bulging anterior fontanelle or neck stiffness), geometric means for those with none of the signs was 0.41 ± 0.08 μg/l, one-to-two signs was 0.42 ± 0.06 μmg/l, three-to-four signs was 1.20 ± 0.49 μg/l and for those with five or more signs was 2.60 ± 2.20 μg/l—Fig. 4. Median PCT level were significantly higher in participants who were admitted 0.42 (0.13–3.47) μg/l compared to those not admitted 0.24 (0.13–0.74) μg/l, *p* = 0.009, Kruskal-Wallis, *χ* ^2^ = 4.4, *p* = 0.04. Children with Syndrome^+ve^ criteria were more likely to be admitted compared to those without clinical syndromic criteria (Syndrome^−ve^), 36/ 49 (73.1%) vs. 63/179 (35.2%), odds ratio = 5.1 (95% CI 2.4–10.7) and had higher proportions with PCT ≥0.5 μg/l—27/49 (55%) vs. 59/179 (33%), odds ratio = 2.5 (95% CI 1.3–4.8).

## Discussion

We aimed to investigate possible bacterial aetiology in children presenting to the emergency department with pneumonia and/or syndromic clinical criteria derived from WHO guidance that are commonly used to target antibiotic prescription using PCT, a biomarker with high specificity for bacterial infection. The study is important because clinical signs alone poorly target children with infection and may result in inappropriate use of antibiotics and attendant risk of antimicrobial resistance [4, 5]. WHO criteria widely used by clinicians in resource poor settings to target antibiotic prescriptions were developed before routine introduction of *H. influenzae* and pneumococcal vaccines and the signs could be less specific due to change in microbial aetiology of infections where viral infections could be more prominent than previously [5, 10, 20]. We found that children with pneumonia or other syndromic clinical criteria used to target antibiotics prescription have higher median PCT levels compared to those without the syndromes. We also found that PCT has criterion validity in this population since higher levels were associated with more severe disease categories. However, PCT levels though higher in severe pneumonia were not significantly different from those in non-severe pneumonia although this may be due to the small sample size. The study hospital is in an area without local transmission of malaria and malaria infection was unlikely in those with elevated PCT levels.

Several studies have shown usefulness of PCT measurement and levels for identifying children for antibiotic prescription, its correlation with severity of disease, and elevated levels in community acquired pneumonia and are consistent with our findings [12, 21–27]. The syndromic criteria on their own lack adequate sensitivity as shown from studies in Kenya [5] and Tanzania [4] and still miss 20–40% of invasive bacteria disease depending on the underlying context. It is therefore possible that children without syndromic criteria who have abnormal PCT levels are those that are missed by the syndromic criteria but we cannot say this with certainty since PCT also lacks adequate specificity and is not a replacement for cultures, the gold standard. Our study is unique because it broadly describes how these syndromic criteria compares to PCT biomarker in this study setting where it has not been done previously. The syndromic criteria are widely used by clinicians in resource poor countries and this is likely to continue because of lack of appropriate diagnostics but with shortcomings of the syndromic criteria and because PCT assay is imperfect, it is difficult to interpret abnormal PCT levels. PCT levels may be useful in monitoring response to treatment since higher levels are associated with more severe disease as found in this study and in previous studies. Use of additional investigations or more clinical signs have been suggested to improve the syndromic criteria [5, 28] but inadequate clinical assessment at hospitals is common in resource poor settings [29–31] and this may not solve the problem and investigations such as lumbar punctures are infrequently done [32]. Clinicians in our study had high level of pre-practice training and were trained in symptom recognition before the study commenced and we believe proper clinical assessments were done.

PCT levels are positively correlated with disease severity but since abnormal levels are seen in even in those without severe disease, this study is unable to recommend antibiotic prescription by PCT levels only. However, because accurate microbiological culture results are unlikely to be widely available soon in resource poor settings, clinical trials on antibiotics guided by PCT levels may be required to assess the place of PCT for guiding antibiotic prescription. If such trials prove efficacious then PCT levels could be considered an empiric criterion for identification of children for antibiotic treatment. While this would not help in tracking antimicrobial resistance, it has potential for improving antibiotic stewardship. The study hospital is private fee-charging institution and serves mainly population of middle-high income earners and this may explain why severe acute malnutrition, a known risk factor of death and bacteraemia, was not seen. Our findings are applicable to settings without stable high malaria transmission and this includes many areas in Kenya [17] and Africa due to the shrinking malaria map and many areas have zero local transmission [33, 34]. Our study was limited because we did not have microbial diagnosis and PCT was only measured once at the time of assessment and this may miss levels that rise later and serial measurements are recommended [35].

## Conclusion

In an area without local malaria transmission, raised serum PCT levels are common in children presenting to emergency department with clinical signs of pneumonia or fulfilling clinical syndromic definitions for suspected bacterial infections and there is an association between elevated serum PCT levels and disease severity. Clinical trials of antibiotic therapy based on PCT assay should be considered in settings where accurate microbiological diagnosis is not available to fully understand its utility for guiding antibiotic therapy.

## Figures and Tables

**Table 1:** Definitions of various clinical syndromes where antibiotics are recommended

Syndrome	Definition
Sick young infants	Hospitalized children aged <60 days
Meningitis/encephalopathy	Neck stiffness, bulging fontanelle or coma
Severe malnutrition	Weight for height Z-score < –3 or kwashiorkor
Very severe pneumonia^a^	Lower chest wall indrawing plus one or more of; inability to sit unassisted if aged ≥ 1 year or inability to drink or breastfeed if aged <1 year, cyanosis or hypoxia (pulse oximetry <90% in air)
Severe pneumonia^b^	Lower chest wall indrawing
Mild pneumonia^b^	Tachypnoea (≥50bpm if aged 60 days to 1 year; ≥40bpm if ≥1-year old) plus a history of either cough or difficulty breathing
Skin or soft tissue infection	Cellulitis, abscess and pyomyositis

a Now referred to as severe pneumonia.

b Both now classified as non-severe pneumonia.

**Table 2:** Characteristics of participants

	Overall	Syndrome^+ve^ criteria (*n* = 49)		Syndrome^−ve^ criteria (*n* = 179)	
		PCT ≥ 0.5 μg/l (*n* = 27)	PCT < 0.5 μg/l (*n* = 22)	PCT ≥ 0.5 μg/l (*n* = 59)	PCT < 0.5 μg/l (*n* = 120)
Female gender, *n* (%)	108 (47)	15 (57)	9 (41)	27 (46)	57 (48)
Age in months, median (IQR)	25 (11–42)	37 (18–44)	13 (7–18)	28 (11–42)	22 (13–44)
Weight, mean ± SD	16.1 ± 8.9	17.1 ± 9.7	15.7 ± 11.9	15.5 ± 7.9	16.3 ± 8.6
Height, mean ± SD	100 ± 23	104 ± 22	95 ± 30	100 ± 25	100 ± 22
Abnormal CRP, *n*/*N* (%)	12/18 (67)	4/4 (100)	1/1 (100)	4/5 (80)	3/8 (38)
Leucocytosis, *n*/*N* (%)	75/193 (39)	16/26 (62)	7/19 (37)	26/52 (50)	26/96 (27)
Leucopoenia, *n*/*N* (%)	5/193 (3)	0/26 (0)	1/19 (5)	0/52 (0)	4/96 (4)
Tachycardia, *n* (%)	55 (24)	11 (41)	6 (27)	9 (52)	29 (24)
Bradycardia, *n* (%)	2 (1)	0 (0)	0 (0)	0 (0)	2 (02)
Tachypnoea, *n* (%)	21 (9)	9 (33)	5 (23)	2 (3)	5 (4)
Admitted, *n* (%)	99 (43)	22 (81)	15 (68)	25 (42)	37 (31)

CRP, C-reactive protein; IQR, interquartile range.
